# Monocytes/Macrophages Upregulate the Hyaluronidase HYAL1 and Adapt Its Subcellular Trafficking to Promote Extracellular Residency upon Differentiation into Osteoclasts

**DOI:** 10.1371/journal.pone.0165004

**Published:** 2016-10-18

**Authors:** Emeline Puissant, Marielle Boonen

**Affiliations:** Laboratoire de Chimie Physiologique - URPhyM, University of Namur, Namur, Belgium; University of Oulu, FINLAND

## Abstract

Osteoclasts are giant bone-resorbing cells originating from monocytes/macrophages. During their differentiation, they overexpress two lysosomal enzymes, cathepsin K and TRAP, which are secreted into the resorption lacuna, an acidified sealed area in contact with bone matrix where bone degradation takes place. Here we report that the acid hydrolase HYAL1, a hyaluronidase able to degrade the glycosaminoglycans hyaluronic acid (HA) and chondroitin sulfate, is also upregulated upon osteoclastogenesis. The mRNA expression and protein level of HYAL1 are markedly increased in osteoclasts differentiated from RAW264.7 mouse macrophages or primary mouse bone marrow monocytes compared to these precursor cells. As a result, the HYAL1-mediated HA hydrolysis ability of osteoclasts is strongly enhanced. Using subcellular fractionation, we demonstrate that HYAL1 proteins are sorted to the osteoclast lysosomes even though, in contrast to cathepsin K and TRAP, HYAL1 is poorly mannose 6-phosphorylated. We reported previously that macrophages secrete HYAL1 proforms by constitutive secretion, and that these are recaptured by the cell surface mannose receptor, processed in endosomes and sorted to lysosomes. Present work highlights that osteoclasts secrete HYAL1 in two ways, through lysosomal exocytosis and constitutive secretion, and that these cells promote the extracellular residency of HYAL1 through downregulation of the mannose receptor. Interestingly, the expression of the other main hyaluronidase, HYAL2, and of lysosomal exoglycosidases involved in HA degradation, does not increase similarly to HYAL1 upon osteoclastogenesis. Taken together, these findings point out the predominant involvement of HYAL1 in bone HA metabolism and perhaps bone remodeling via the resorption lacuna.

## Introduction

Osteoclasts are giant multinucleated cells responsible for bone resorption that differentiate from hematopoietic cells of monocyte/macrophage lineage. When in contact with bone, osteoclasts polarize and form a resorption lacuna sealed by an actin ring in which the bone matrix is degraded, notably by secreted lysosomal acid hydrolases [1,2]. These are released when secretory lysosomes fuse with the osteoclast apical membrane, a process that also provides vATPase complexes which generate an acidified extracellular environment optimal for lysosomal hydrolase activity [3–7]. Secretory lysosomes of osteoclasts are particularly enriched in two lysosomal hydrolases: the cysteine protease cathepsin K and the acid phosphatase TRAP (Tartrate-Resistant Acid Phosphatase). Exocytosis of the lysosomal content appears to be the primary mechanism underlying the secretion of cathepsin K (under its activated/processed form), whereas TRAP can be secreted in two ways: by exocytosis and, under its precursor form, by the constitutive secretory pathway [7,8]. Cathepsin K degrades type I collagen, a major constituent of bone matrix [9,10], but the function of TRAP is less well understood. Except in brain, TRAP is responsible for the dephosphorylation of acid hydrolases upon their arrival in the lysosomal compartment [11]. In the bone system, it has been suggested that TRAP regulates the migration of osteoclasts through dephosphorylation of osteopontin, a protein involved in osteoclast adhesion to bone matrix [12]. The ability of TRAP to generate radical oxygen species might also contribute to the degradation of bone matrix products transcytosed from the resorption lacuna [13,14]. Cathepsin K or TRAP deficient mice both exhibit osteopetrosis, an increase of bone mineral density, demonstrating that these enzymes are required to maintain bone homeostasis [15,16]. Interestingly, one common feature of cathepsin K and TRAP is their marked upregulation, in contrast to other lysosomal acid hydrolases, upon differentiation of osteoclasts from their monocytic precursors [17].

Several studies have highlighted that hyaluronic acid (HA), a high molecular mass (MM) glycosaminoglycan present in extracellular matrices, including bone matrix, can influence the differentiation and activity of osteoclasts in a size-dependent manner. Whereas low MM HA can stimulate the differentiation and bone resorption activity of murine and human osteoclasts, high MM HA exhibits the opposite effect [18–21]. Of note, the MM profile of HA in various somatic tissues can be modulated through the hydrolytic action of two main endoglycosidases, the hyaluronidases HYAL1 and HYAL2, aided by two lysosomal exoglycosidases, β-hexosaminidase and β-glucuronidase. A model of HA catabolism [22] proposes that HYAL2, a glycosylphosphatidylinositol-anchored cell surface hyaluronidase [23], starts to cleave high MM HA into smaller fragments, probably down to 20 kDa [24]. These fragments are then degraded more thoroughly by HYAL1 and exoglycosidases in the endo/lysosomal system. Whether a similar system exists in the bone matrix is unknown.

We have now explored the hyaluronidases of osteoclasts and report that the expression of HYAL1, but not HYAL2, is strongly upregulated, at the mRNA and protein level, upon *in vitro* differentiation of macrophages into osteoclasts. In addition, our study reveals that the osteoclast differentiation process includes changes in the subcellular trafficking of HYAL1 that promote its extracellular residency, thereby providing new insight into bone metabolism.

## Results

### The expression level of HYAL1 increases strikingly during osteoclastogenesis

**Fig 1:**
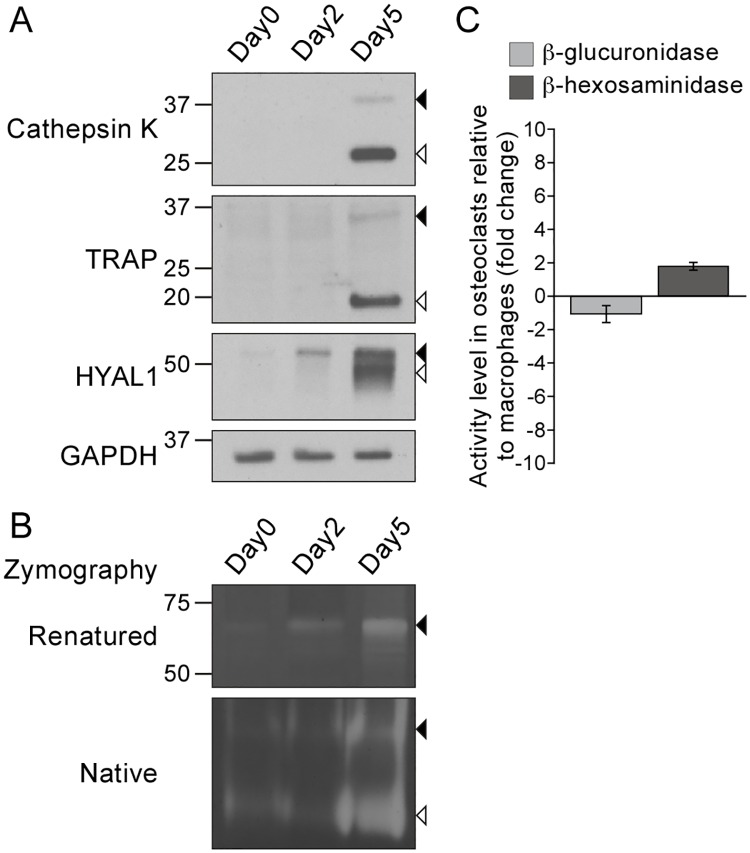
The protein and activity levels of HYAL1 are upregulated during osteoclastogenesis. RAW264.7 macrophages were treated for 2 or 5 days with RANKL to induce osteoclastogenesis. (A) Detection of cathepsin K, TRAP, HYAL1 and GAPDH by western blotting (under reducing conditions). Closed and open arrowheads point to each hydrolase precursor and mature form, respectively. (B) Visualization of the HA-degrading activity of HYAL1 by renatured protein zymography (“Renatured”) and native protein zymography (“Native”) (non-reducing conditions). Precursor and mature forms are pointed as described in A. (C) Measurement of the activity of β-hexosaminidase and β-glucuronidase in RAW264.7 macrophages and in osteoclasts derived from these cells. The graph shows the fold change (mean ± SD of *n* = 3 independent experiments) in osteoclasts relative to the precursor cells.

To investigate the expression of HA-degrading enzymes during osteoclastogenesis, we used a well-established *in vitro* system, i.e. the differentiation of RAW264.7 macrophages by treatment with the receptor activator of nuclear factor kappa-B ligand (RANKL), a cytokine that induces osteoclastogenesis through binding to its receptor RANK located at the surface of monocytes/macrophages [25]. As expected (Fig 1A), we observed that differentiated osteoclasts (i.e. treated with RANKL for 5 days) contain large amounts of mature/proteolytically processed forms of cathepsin K and TRAP (Fig 1A, open arrowheads), as well as low amounts of their precursor forms (closed arrowheads), whereas macrophages (day 0) and cells treated for 2 days with RANKL are mostly devoid of these proteins. We previously reported that RAW264.7 macrophages contain two main forms of HYAL1 that are detected by western blotting under reducing conditions: a ~52 kDa precursor form that bears high-mannose *N*-linked glycans and likely localizes to the endoplasmic reticulum (ER), and a mature form of ~48 kDa produced by proteolytic cleavage [26]. The precursor is processed during transport of HYAL1 through endosomes, on its way to lysosomes where the mature form accumulates [26]. Interestingly, we detected an increased level of the 52 kDa precursor form of HYAL1 (closed arrowhead) in osteoclasts as early as day 2 of the differentiation process, and a strong upregulation of both precursor and mature forms at day 5 (Fig 1A), suggesting that differentiated osteoclasts may contain large amounts of HYAL1 in their endo/lysosomal system. Of note, HYAL1 could also be detected in macrophages (day 0) using a longer exposure time (data not shown), in accordance with published findings [26]. Based on the semi-quantitative analysis of 6 independent western blotting experiments, we estimated that the population of HYAL1 proteins is increased by 25.5 ± 6.4-fold in differentiated osteoclasts (day 5) compared to precursor cells (day 0) (p<0.001, non-paired Student’s t-test using the housekeeping protein GAPDH for comparison).

To test whether, as a consequence of HYAL1 upregulation, osteoclasts become more proficient in HA degradation, we used an in-gel HA degradation assay that specifically allows visualization of HYAL1-mediated HA hydrolysis [26–30]. This zymography can be run either in a non-denaturating polyacrylamide gel (referred to as “native protein” zymography) or after separation of the proteins in a SDS-containing gel, followed by a renaturation step (referred to as “renatured protein” zymography). We have recently demonstrated that the precursor form of HYAL1 can degrade HA in both native and renatured protein zymography assays, whereas the activity of the cleaved/lysosomal form of murine HYAL1 can be efficiently detected by native protein zymography only, suggesting that non-covalent interactions support the activity of the latter [26,31]. In accordance with these prior findings, analysis of RAW264.7 macrophage and osteoclast samples by renatured protein zymography revealed a single HA degradation area around 70 kDa at day 2 and 5 of the differentiation process, which is where the precursor form of HYAL1 migrates under non-reducing conditions (Fig 1B, upper panel, closed arrowhead). Moreover, this form exhibited the same expression profile than the 52 kDa precursor form of HYAL1 detected by western blotting (reducing conditions, Fig 1A). HA degradation by this proform was also detected by native zymography (Fig 1B, lower panel, closed arrowhead), in addition to a larger area devoid of HA lower in the gel, most prominent at day 5 of differentiation (Fig 1B, lower panel, open arrowhead). Previous findings [26] and comparison with the signal intensities obtained by western blotting (Fig 1A), identify this signal as the result of HA degradation by the mature form of HYAL1. Taken together, these observations demonstrate that upregulation of HYAL1 proteins in osteoclasts significantly enhances their HA degradation ability compared to the precursor cells.

Of note, these findings were validated using osteoclasts differentiated from primary mouse bone marrow monocytes/macrophages (BMM). S1 Fig shows that the differentiation of these cells in the presence of RANKL and M-CSF (macrophage colony-stimulating factor, added to support BMM proliferation, differentiation and survival [32]) results in a similar increase of HYAL1 protein and activity levels in osteoclasts. This supplemental figure also includes a control of the specificity of the anti-HYAL1 antibody.

### HYAL1 is the only HA-degrading enzyme upregulated in osteoclasts

We used quantitative PCR (qPCR) to measure the mRNA levels of HA endo- and exo-glycosidases. These analyses revealed a 4.0-fold increase of *Hyal1* mRNA level 2 days after addition of RANKL, and a 11.1-fold increase when osteoclasts were fully differentiated (Table 1), suggesting that HYAL1 upregulation upon osteoclast differentiation is likely accounted for, at least partly, by an enhanced transcription. Indeed, this progressive upregulation, albeit not as striking as the increases measured for cathepsin K and TRAP encoding mRNAs (Table 1), nicely correlates with the elevation of HYAL1 protein and activity levels that occurs upon osteoclast differentiation (Fig 1A and 1B).

**Table 1:** Relative mRNA expression levels of hyaluronidases and lysosomal hydrolases in osteoclasts collected at day 2 or 5 of the differentiation process, compared to RAW264.7 precursor macrophages (day 0).

Protein / Gene name	Fold change	
	Day 2 / Day 0	Day 5 / Day 0
**Cathepsin K / *Ctsk***	32.80	296.11
**TRAP / *Acp5***	154.20	534.85
**HYAL1 / *Hyal1***	4.01	11.10
**HYAL2 / *Hyal2***		1.30
**HYAL3 / *Hyal3***		7.67
**β-glucuronidase / *Gusb***		-1.14
**β-hexosaminidase α / *Hexa***		2.63
**β-hexosaminidase β / *Hexb***		4.58
**Cathepsin D / *Ctsd***		1.08
**β-galactosidase / *Glb1***		3.72
**β-glucocerebrosidase / *Gba***		1.04
**β-mannosidase / *Manba***		1.72

By contrast, the relative amounts of mRNAs coding for HYAL2 (the other active hyaluronidase of somatic tissues) and for the exoglycosidase β-glucuronidase remained relatively stable upon differentiation (Table 1). 2.6-fold and 4.6-fold increases were detected for β-hexosaminidase encoding mRNAs (α and β subunits, respectively). However, only marginal changes of activity levels were detected upon osteoclastogenesis for both HA exoglycosidases using *in vitro* enzyme assays (Fig 1C): a 1.1 ± 0.5-fold decrease was measured for β-glucuronidase and a 1.8 ± 0.2-fold increase for β-hexosaminidase (p = 0.45 and p<0.01, respectively; non-paired Student’s t-tests conducted on *n* = 3 independent experiments using GAPDH as a housekeeping protein). A larger elevation of 7.7-fold was measured for the mRNA coding for HYAL3. This observation is not surprising as co-transcription of HYAL1 and HYAL3 encoding genes, which are tightly clustered, has been reported in mouse liver [33]. Albeit its role in HA hydrolysis remains elusive, as this protein appears to have no HA degradation activity in somatic cells [29,34], HYAL3 may increase the expression and activity of HYAL1 [29]. Of note, in accordance with published findings [17], the mRNA expression level of several other acid hydrolases (including cathepsin D, β-galactosidase, β-glucocerebrosidase and β-mannosidase) only exhibited limited variations compared to HYAL1, cathepsin K and TRAP (Table 1), confirming that only a subset of acid hydrolases are upregulated upon osteoclastogenesis. Similar observations were made in BMM-derived osteoclasts (S1 Table and S1D Fig).

Taken together, these findings suggest that HYAL1 likely plays a predominant role in osteoclasts, compared to other acid hydrolases and HA-degrading enzymes.

### The processed form of HYAL1 localizes to lysosomes in osteoclasts

**Fig 2:**
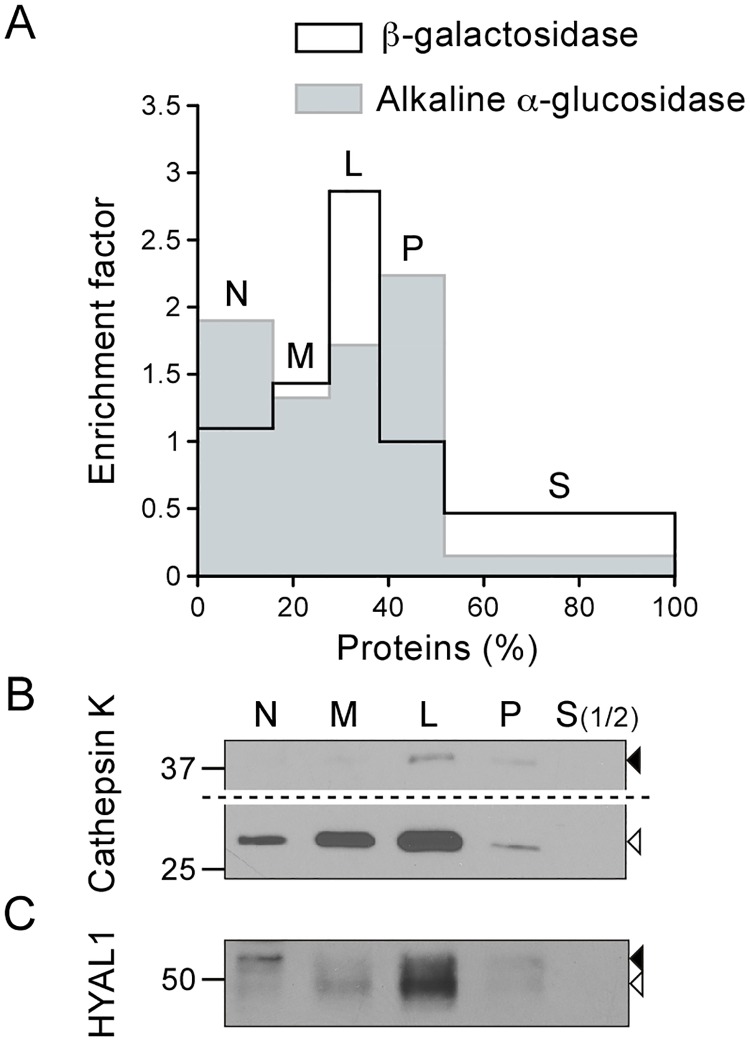
The mature form of HYAL1 co-distributes with lysosomal markers after osteoclast fractionation by differential centrifugation. Osteoclasts differentiated from RAW264.7 cells were fractionated into five fractions (N, M, L, P and S) following de Duve's fractionation scheme. (A) The activities of β-galactosidase and alkaline α-glucosidase were detected by fluorometric assay to establish the distribution of lysosomes and of the ER, respectively. The graph shows the relative specific activity (ratio of the percentage of activity of the enzyme in a given fraction to the percentage of proteins in this fraction), which is indicative of the enrichment factor of the enzyme in the fractions, plotted against the percentage of proteins in each fraction. (B-C) The distribution of cathepsin K and HYAL1 was analyzed by western blotting (reducing conditions). Equal amounts of proteins were loaded for each fraction, except for S, which was diluted 1:2 compared to the other fractions. The mature and precursor forms are highlighted by open and closed arrowheads, respectively. Of note, in panel B, a longer exposure time is shown for the upper part of the blot to help visualization of cathepsin K proforms.

Our previous study of the subcellular trafficking of HYAL1 in RAW264.7 cells demonstrated that the mature form of HYAL1 accumulates in lysosomes where it can exert its activity [26]. To investigate the intracellular localization of HYAL1 in osteoclasts, we fractionated RAW264.7-derived osteoclasts into 5 fractions by differential centrifugation, i.e. nuclear (N), heavy mitochondrial (M), light mitochondrial (L), microsomal (P), and cytosolic (S) [35]. As expected, the lysosomal marker enzyme β-galactosidase (detected by enzyme assay, Fig 2A) and the mature form of cathepsin K (detected by western blotting, Fig 2B, open arrowhead) were found predominantly enriched in the L fraction, and to a lesser extent in the M fraction. In contrast, the precursor form of cathepsin K (Fig 2B, closed arrowhead) distributed between the L and P fractions, which is consistent with its presence in pre-lysosomal compartments. Indeed, the P fraction is more enriched in biosynthetic compartments such as the ER, compared to the M and L fractions, as indicated by the distribution profile of the ER marker alkaline α-glucosidase (Fig 2A). Similarly to the cathepsin K proform, the 52 kDa HYAL1 precursor form distributed between the L and P fractions (Fig 2C, closed arrowhead). In addition, the mature form of HYAL1 (Fig 2C, open arrowhead) was found enriched in the M and L fractions, as observed for the lysosomal markers (β-galactosidase and mature cathepsin K).

**Fig 3:**
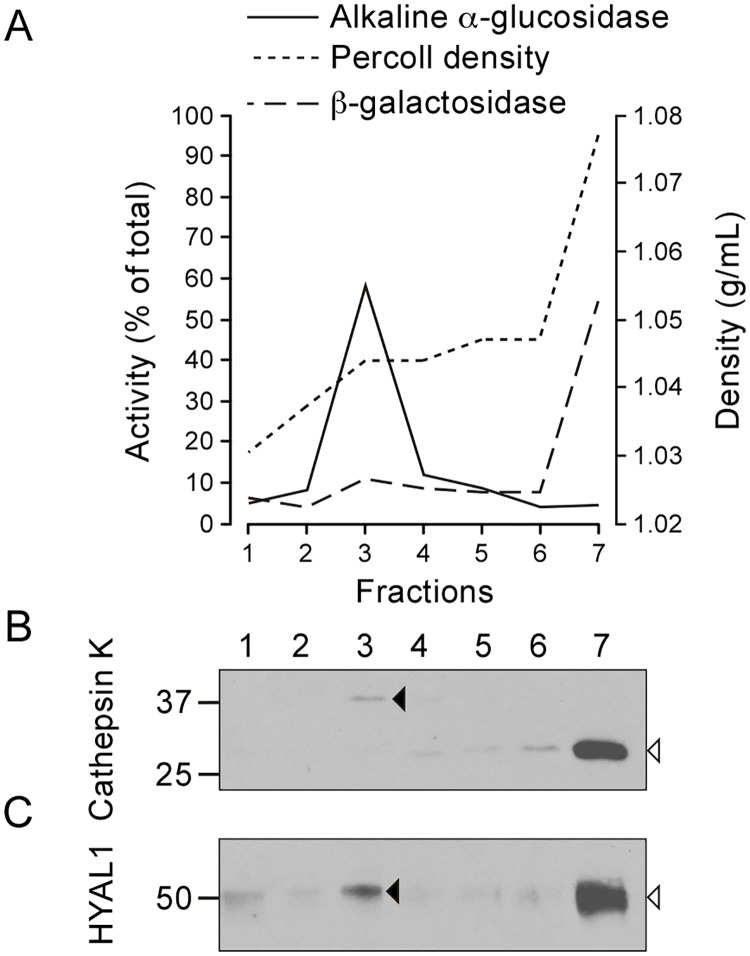
HYAL1 and cathepsin K co-distribute in a self-forming Percoll^™^ density gradient. An M+L+P pooled fraction of osteoclasts was centrifuged in a self-forming Percoll^™^ density gradient and 7 fractions were collected from top to bottom. (A) The distribution of lysosomes and of the ER was established based on the activities of β-galactosidase and alkaline α-glucosidase, respectively. The graph shows the percentage of β-galactosidase and alkaline α-glucosidase in each fraction of the Percoll^™^ gradient and the density of these fractions. (B-C) The distribution of the precursor and mature forms of cathepsin K and HYAL1 (pointed by closed and open arrowheads, respectively) was analyzed by western blotting (reducing conditions).

To test whether these co-distributions reflected the presence of HYAL1 precursor forms in pre-lysosomal structures and of mature forms in lysosomes, we used two different density gradient centrifugation methods. Of note, we could not use an immunofluorescence assay as non-specific staining is detected with currently available anti-HYAL1 antibodies. Hence, we prepared a pooled M+L+P fraction, which was centrifuged in a self-forming Percoll^™^ density gradient, allowing lysosomes (enriched in β-galactosidase and mature cathepsin K) to sediment until the densest fraction of the gradient. In contrast, biosynthetic/pre-lysosomal compartments (containing alkaline α-glucosidase and the cathepsin K precursor) remained in the lower density zone (Fig 3A and 3B). Detection of HYAL1 by western blotting revealed the presence of the 52 kDa precursor form of HYAL1 in the upper region of the gradient (Fig 3C, closed arrowhead). This is in accordance with its localization in pre-lysosomal compartments. By contrast, the mature form of HYAL1 was detected in the lysosome-containing fraction at the bottom of the gradient (Fig 3C, open arrowhead).

**Fig 4:**
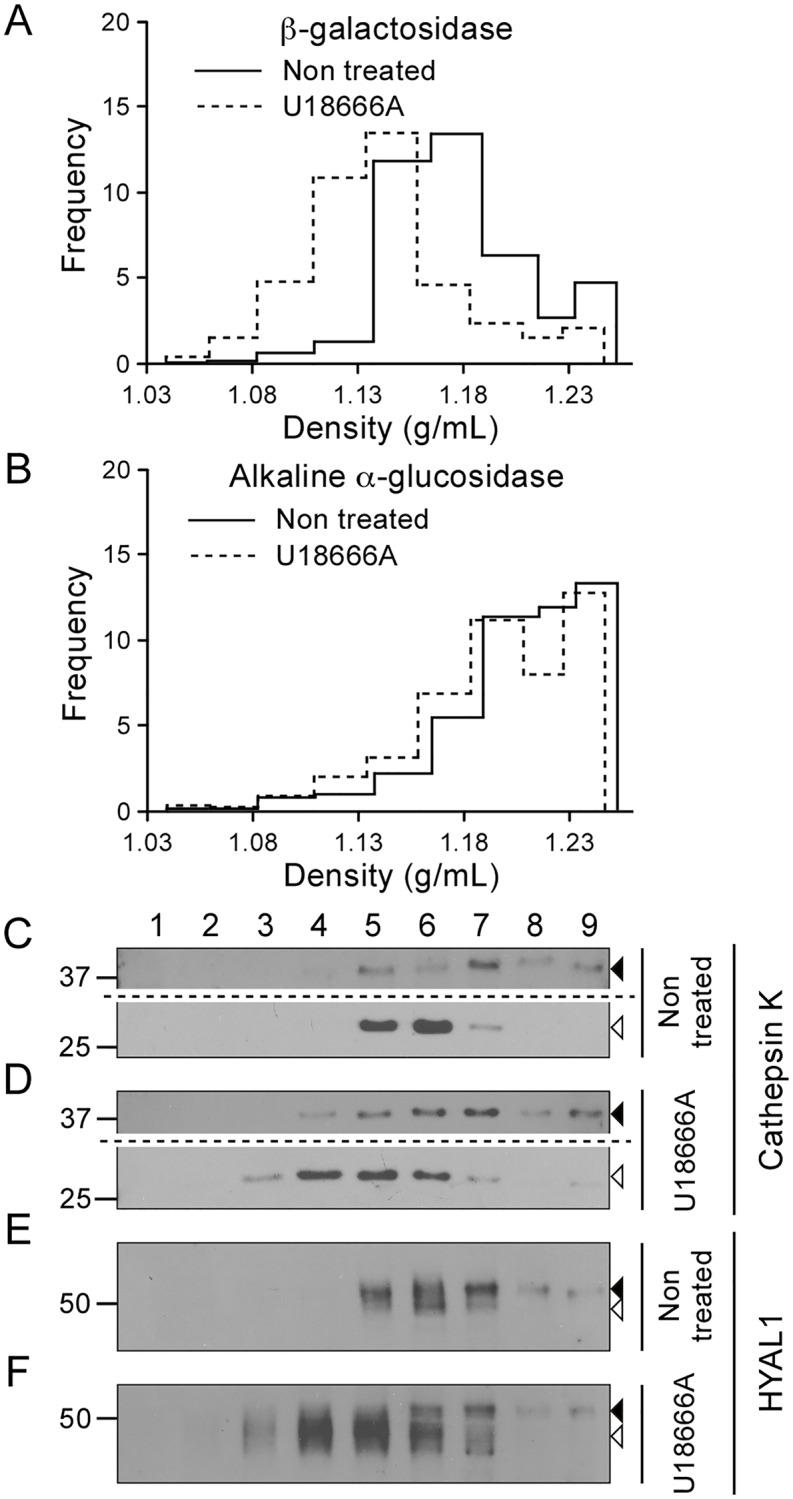
U18666A modifies the distribution of mature HYAL1 in a sucrose gradient, similarly to lysosomal markers. An M+L+P fraction was prepared from control or U18666A treated osteoclasts and centrifuged in a linear sucrose density gradient extending from 1.04 g/mL to 1.26 g/mL. Nine fractions were collected from top to bottom. (A-B) The activities of the markers β-galactosidase (A) and alkaline α-glucosidase (B) were measured using fluorometric assay in the different fractions. The ordinate of the graphs corresponds to the frequency (Q/SQ.r, where Q represents the activity found in the fraction, SQ, the total activity recovered in the sum of the fractions, and r, the increment of density from top to bottom of the fraction). (C-F) Cathepsin K and HYAL1 were detected by western blotting (reducing conditions) in the fractions collected after centrifugation. The precursor and mature forms of these proteins are shown by closed and open arrowheads, respectively. Of note, a longer exposure time is shown for the upper part of the blots shown in panels C and D, to visualize cathepsin K proforms.

Lastly, an M+L+P pooled fraction was prepared from control osteoclasts or osteoclasts treated for 24 h with U18666A, and fractionated in a linear sucrose density gradient. U18666A is a chemical compound that inhibits cholesterol egress from lysosomes and, as a result, modifies the density of lysosomes in sucrose gradients [36]. This was reflected here by a shift of distribution of β-galactosidase and of mature cathepsin K toward the lower density region of the gradient (Fig 4A, 4C and 4D). Similarly, the mature form of HYAL1, which co-distributed with β-galactosidase and mature cathepsin K in control conditions, was detected in lower density fractions after treatment (Fig 4E and 4F). Importantly, the distributions of the precursor forms of HYAL1 and cathepsin K, and of the ER marker alkaline α-glucosidase were only marginally, if at all, affected by the U18666A treatment demonstrating that the density shift is specific to lysosomes (Fig 4B–4F).

Taken together, these three fractionation experiments results demonstrate the lysosomal residency of large amounts of mature HYAL1 in osteoclasts while the precursor form is mostly present in pre-lysosomal compartments (e.g. the ER).

### Osteoclasts secrete HYAL1 by exocytosis of lysosomes and constitutive secretion

**Fig 5:**
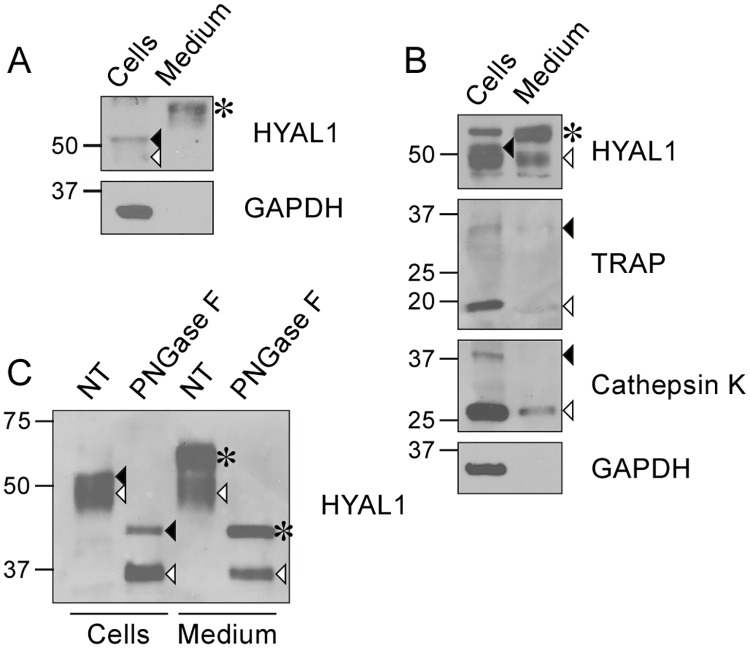
Osteoclasts secrete HYAL1 through both the constitutive secretory pathway and lysosomal exocytosis. (A) Detection of HYAL1 by western blotting, under reducing conditions, in RAW264.7 cell lysate and medium samples collected after a 5 h incubation in serum-free conditions. The cytosolic protein GAPDH was used as a control of the cell integrity. The medium was concentrated 4-fold compared to the cell sample. The closed arrowhead, open arrowhead and asterisk mark the 52 kDa intracellular precursor form, 48 kDa intracellular mature form and 65 kDa secreted form of HYAL1, respectively. (B) Detection of cathepsin K, TRAP, GAPDH and HYAL1 by western blotting in RAW264.7-derived osteoclast cell lysate and concentrated medium (4 x). The precursor and mature forms of TRAP and cathepsin K are highlighted by closed and open arrowheads, respectively. The different forms of HYAL1 are marked as described in A. (C) Aliquots of osteoclast cell lysate and medium (concentrated 4-fold) were treated with PNGase F prior to the detection of HYAL1 by western blotting. The different forms of HYAL1 are pointed as described in A. Note that the extracellular precursor form (asterisk) exhibited the same MM as the intracellular precursor (closed arrowhead) after deglycosylation, and that osteoclasts secreted mature forms of HYAL1 (detected at ~35 kDa after deglycosylation, open arrowhead).

We previously reported that RAW264.7 macrophages secrete a single form of HYAL1 of ~65 kDa via the constitutive secretory pathway [26], i.e. a precursor form that traffics through the Golgi apparatus where its *N*-linked oligosaccharidic chains become of the complex-type, giving rise to a slightly higher form compared to the intracellular, newly synthesized 52 kDa precursor that bears high-mannose glycans (Fig 5A, see asterisk and closed arrowhead, respectively). After deglycosylation with Peptide-*N*-Glycosidase F (PNGase F), which removes all *N*-linked glycans from glycoproteins, both intracellular (52 kDa) and secreted (65 kDa) precursor forms exhibit the same MM of ~45 kDa, whereas the 48 kDa mature form of HYAL1 (exclusively intracellular, open arrowhead in Fig 5A) exhibits a protein backbone of ~35 kDa after deglycosylation, reflecting its proteolytic processing in endo/lysosomes. The results of these glycosidase treatments can be found in Puissant et al. [26].

Interestingly, in contrast to macrophages, we found that osteoclasts derived from RAW264.7 cells secrete two forms of HYAL1, i.e. the 65 kDa complex glycan-bearing form (Fig 5B, asterisk) and the 48 kDa processed form (Fig 5B, open arrowhead). Indeed, treatment of osteoclast medium with PNGase F showed that the two secreted HYAL1 forms exhibited MM of ~45 and ~35 kDa after complete *N*-deglycosylation, respectively (Fig 5C). Since there was no loss of cellular integrity (GAPDH, a cytosolic protein, was absent from the medium), and since the neutral extracellular pH is not favorable to HYAL1 maturation (as osteoclasts do not form an acidified lacuna when cultured on plastic), it is likely that some amount of HYAL1 transited through lysosomes prior to secretion. In support of this view, the mature forms of TRAP and cathepsin K could also be detected in the culture medium of osteoclasts (Fig 5B), and these results were confirmed using BMM-derived osteoclasts (S1E Fig). Thus lysosomal exocytosis and the constitutive secretory pathway both contribute to the release of large amounts of HYAL1 in the culture medium of osteoclasts.

### The recapture of secreted HYAL1 is downregulated in osteoclasts compared to precursor macrophages

Most lysosomal hydrolases use the mannose 6-phosphate (Man-6-P)-dependent pathway to travel to lysosomes. They receive Man-6-P residues on their *N*-linked glycans during passage through the Golgi apparatus, which allows their recognition by Man-6-P receptors (MPRs) and subsequent packaging into transport vesicles that travel from the *trans*-Golgi network and/or the plasma membrane to endo/lysosomes [37,38]. In osteoclasts, both cathepsin K and TRAP use this pathway to travel to secretory lysosomes [7,17]. In contrast, the intracellular trafficking of cathepsin D is independent of Man-6-P in osteoclasts, indicating that there are also other ways to travel to lysosomes in those cells [7]. In RAW264.7 macrophages, HYAL1 is targeted to lysosomes by a Man-6-P-independent secretion/recapture mechanism involving endocytosis by the cell surface mannose receptor [26]. Similarly, it has been reported recently that 22Rv1 human prostate adenocarcinoma cells secrete and recapture HYAL1 by endocytosis, and that this process modulates HA internalization [39]. As bone matrix resorption relies on the concentration of acid hydrolases in an extracellular degradation lacuna, we wondered whether HYAL1, which is largely secreted by osteoclasts (see above), was also sorted to lysosomes by Man-6-P-independent recapture from the extracellular medium, a mechanism that would seem rather counterproductive in this context.

**Fig 6:**
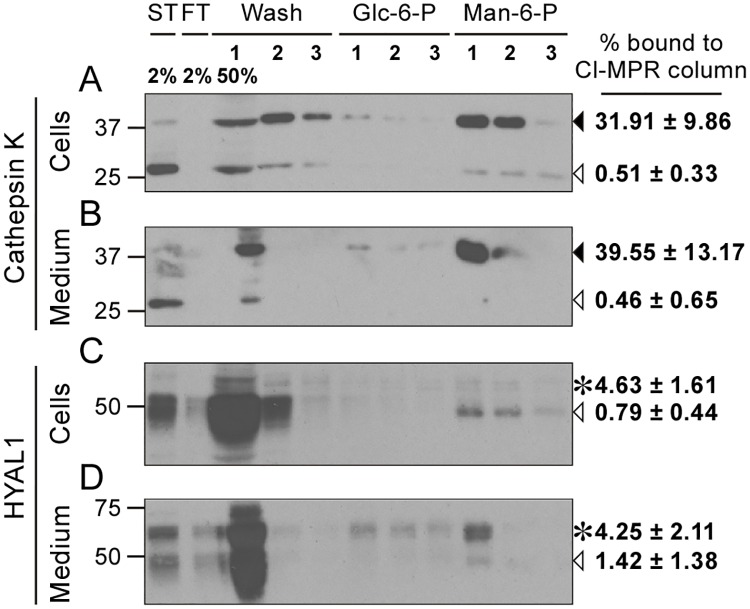
HYAL1 is poorly mannose 6-phosphorylated in osteoclasts. Cell lysate (A, C) or culture medium (B, D) of osteoclasts differentiated from RAW264.7 cells were collected after a 5 h culture period in serum-free conditions and loaded on a CI-MPR affinity column. The presence of cathepsin K (A-B) and HYAL1 (C-D) was analyzed, using western blotting (reducing conditions), in the starting sample (ST), flow-through (FT), washes (Wash) and elution fractions (trichloroacetic acid-precipitated). Proteins non specifically bound to the column were eluted using 5 mM of Glucose 6-Phosphate (Glc-6-P) while proteins that bound specifically to CI-MPRs were eluted with 5 mM of mannose 6-phosphate (Man-6-P). Cathepsin K precursor and cleaved forms are indicated by closed and open arrowheads, respectively. The 65 kDa precursor and 48 kDa mature forms of HYAL1 are indicated by asterisks and open arrowheads, respectively. One representative experiment is shown. Specific binding of each cathepsin K or HYAL1 forms to the column was calculated in *n* = 3 independent experiments and expressed as a percentage of the total signal detected for the corresponding form in the starting sample (mean ± SD of the 3 independent experiments are shown).

First, we investigated the mannose-6-phosphorylation level of HYAL1 in osteoclasts using a Man-6-P receptor (CI-MPR) affinity column, as previously described [26,40]. As a control, we measured the mannose 6-phosphorylation level of cathepsin K, which uses this pathway to travel to lysosomes in those cells [7,17]. In accordance with the presence of TRAP in osteoclast lysosomes, which removes Man-6-P signals on acid hydrolases [11], the binding of the intracellular and secreted mature forms of cathepsin K to the column were very low. Only 0.5 ± 0.3% and 0.5 ± 0.7% of the total mature forms eluted after addition of Man-6-P, respectively (Fig 6A and 6B, open arrowheads). In contrast, 31.9 ± 9.9% of the intracellular precursor form of cathepsin K and 39.6 ± 13.2% of its secreted precursor form (Fig 6A and 6B, closed arrowheads) bound to the column, confirming that newly synthesized cathepsin K is efficiently phosphorylated. When the analysis was performed on HYAL1, we observed that, similarly to cathepsin K, only marginal amounts of mature/lysosomal HYAL1 bound to the column: 0.8 ± 0.4% of intracellular mature HYAL1 and 1.4 ± 1.4% of the mature form secreted by lysosomal exocytosis (Fig 6C and 6D, open arrowheads). However, in contrast to cathepsin K, the phosphorylation level of HYAL1 was also very low on its precursor form: only 4.6 ± 1.6% and 4.3 ± 2.1% of the intracellular and secreted 65 kDa precursor forms bore Man-6-P signals, respectively (Fig 6C and 6D, asterisks). Binding of the intracellular 52 kDa precursor forms to the column was not detected. The slightly higher phosphorylation level of the 65 kDa HYAL1 precursor forms (compared to the 48 kDa mature forms) is consistent with some HYAL1 precursor forms exiting the cells through the constitutive secretory pathway, bypassing TRAP-containing lysosomes, but also suggests that HYAL1 is not a good substrate of the phosphotransferase that catalyzes the first step of Man-6-P synthesis. Overall, the average phosphorylation level of the HYAL1 precursor in osteoclasts only amounted to 4.4 ± 1.8%, compared to 35.7 ± 11.2% for cathepsin K (non-paired Student’s t-test, *n* = 3 independent experiments, p<0.001). This finding indicates that, similarly to macrophages, osteoclasts mostly target HYAL1 to lysosomes by Man-6-P-independent pathway(s), and points out that HYAL1 secreted by osteoclasts is only weakly, if at all, recaptured by Man-6-P receptors.

**Fig 7:**
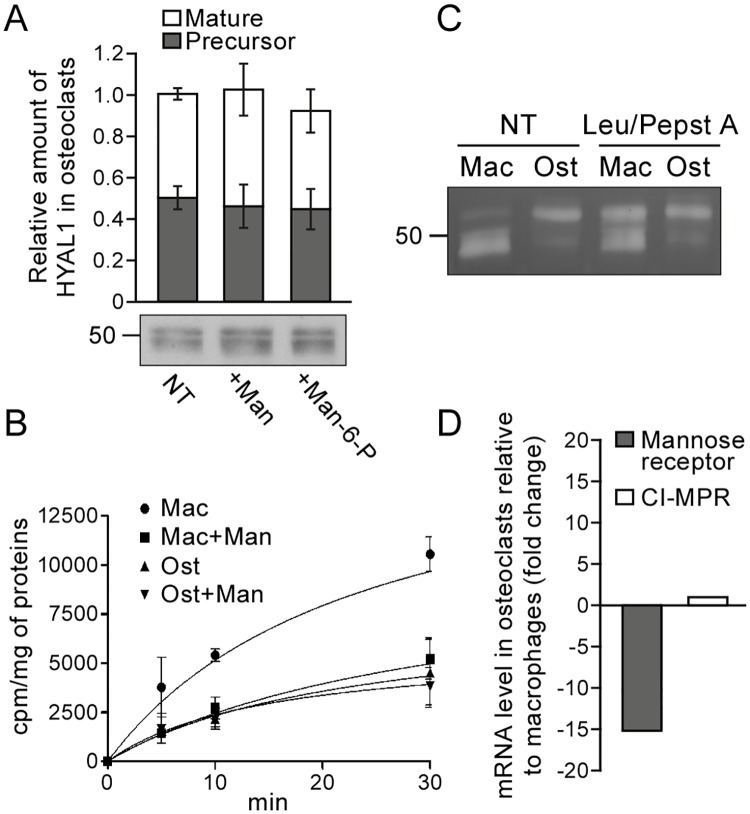
Osteoclasts downregulate the mannose receptor-dependent recapture mechanism that targets HYAL1 to lysosomes in macrophages. (A) Osteoclasts derived from RAW264.7 cells were treated for 24 h with 15 mg/mL of mannan (Man) or 5 mM of Man-6-P, and the intracellular amount of HYAL1 was subsequently visualized using western blotting. The graph shows the quantifications of 52 kDa precursor (grey squares) and 48 kDa mature (white squares) HYAL1 forms in *n* = 3 independent experiments (mean ± SD). (B) Osteoclasts (Ost) and their RAW264.7 precursor macrophages (Mac) were incubated for 5, 10 or 30 min with iodinated rhHYAL1. When indicated, 10 mg/mL of mannan (Man) were added on the cells 5 min prior to the assay and 20 mg/mL during the pulse period. After washing, the proteins bound to the surface were stripped prior to cell lysis and counting of the internalized radioactivity. Three independent experiments were quantified (mean ± SD). (C) After a 2 h incubation at 37°C with rhHYAL1 (± leupeptin [Leu]/pepstatin A [Pepst A]) followed by a 15 min chase, the amount of rhHYAL1 endocytosed by macrophages (Mac) and osteoclasts (Ost) was detected using renatured protein zymography. Of note, only low amounts of proteins are loaded on the gel in these assays; therefore, the endogenous protein is below detection level. One representative experiment is shown (total independent experiments *n* = 3). (D) The mRNA expression levels of the mannose receptor and CI-MPR were measured by qPCR in macrophages and osteoclasts. The ΔCT values obtained in 3 independent experiments were averaged and used to calculate fold changes in osteoclasts relative to precursor macrophages (2^-ΔΔCT^).

Next, we tested whether the mannose receptor recaptured secreted HYAL1 in osteoclasts. Previously we showed that the addition of mannan, a mannose receptor ligand, to the culture medium of RAW264.7 cells for 24 h significantly reduces the endocytosis of HYAL1 and, as a consequence, the intracellular level of mature/lysosomal HYAL1 which originates from the recapture of the secreted protein [26]. However, in osteoclasts, no change of HYAL1 intracellular level was detected after a 24 h incubation period in the presence of mannan, or Man-6-P for that matter (Fig 7A, no statistically significant differences between groups as determined by a one-way ANOVA analysis, using a Bonferroni’s post-test for multiple comparisons, *n* = 3 independent experiments). Interestingly, an endocytosis assay conducted using ^125^I-labeled recombinant human (rh) HYAL1 revealed that osteoclasts internalize 2.3 ± 0.1, 2.6 ± 0.5 and 2.6 ± 1.2 times less enzyme than macrophages after 5, 10 and 30 min of incubation at 37°C (Fig 7B, p<0.05, 0.001 or 0.01, respectively. Non-paired Student’s t-tests, *n* = 3 independent experiments). Moreover, whereas mannan efficiently inhibited the capture of ^125^I-HYAL1 by macrophages (as reported previously [26]), it had no impact on HYAL1 internalization in osteoclasts (Fig 7B). These results suggest that the secretion/recapture mechanism identified in macrophages may not be the main trafficking route followed by HYAL1 to reach lysosomes in osteoclasts. In accordance with this hypothesis, efficient sorting of endocytosed rhHYAL1 to lysosomes was only observed in macrophages. Indeed, using a renatured protein zymography assay, we estimated that only 22.9 ± 4.5% of rhHYAL1 endocytosed by osteoclasts was converted into mature forms after a 2 h chase period, whereas this percentage reached 91.3 ± 1.9% in macrophages (p<0.001, non-paired Student’s t-test, *n* = 3 independent experiments). Of note, knowing that the maturation process of endocytosed rhHYAL1 (i.e. its transport to lysosomes) progressively causes a loss of its activity in the renatured protein zymography assay [26], we also conducted this internalization assay in the presence of protease inhibitors to prevent HYAL1 maturation. This translated by an increase of approximately 35.3 ± 5.1% of the total signal detected in macrophages compared to non-treated macrophages (p<0.01, non-paired Student’s t-test, *n* = 3 independent experiments) and, as expected, in a larger proportion of HYAL1 detected under precursor form (Fig 7C). However, no changes of signal intensities or ratio of HYAL1 forms occurred in treated osteoclasts versus non-treated osteoclasts (non significant differences, non-paired Student’s t-test, *n* = 3 independent experiments), further supporting that very little amount of the endocytosed rhHYAL1 reaches lysosomes in those cells. Coupled with the lower capture efficiency of HYAL1 by osteoclasts (Fig 7B), these results support that recapture of secreted HYAL1 is an inefficient pathway in osteoclasts. Interestingly, we correlated this finding with a large decrease of expression of the mannose receptor in differentiated osteoclasts. qPCR analyses revealed a 15.1-fold decrease of mannose receptor encoding mRNA level in osteoclasts compared to RAW264.7 precursor cells. For comparison, we also measured the mRNA level coding for the CI-MPR: it was unchanged after differentiation of the macrophages into osteoclasts (Fig 7D).

Taken together, our results support that: 1) HYAL1 is mainly sorted to lysosomes in osteoclasts by an intracellular Man-6-P-independent pathway; 2) osteoclasts secrete large amounts of HYAL1 through constitutive secretion and lysosomal exocytosis; and 3) osteoclasts promote the accumulation of HYAL1 in the extracellular space (and probably the resorption lacunae) by downregulating its recapture through the mannose receptor.

## Discussion

In this study, we report that the mRNA expression, protein level and activity of the hyaluronidase HYAL1 are highly upregulated upon osteoclastogenesis. Furthermore, we demonstrate that HYAL1 resides in lysosomes in osteoclasts and that these cells secrete both the precursor form of HYAL1 (likely exiting the cells via the constitutive secretory pathway) and its mature/cleaved form by lysosomal exocytosis. Although HYAL1 also localizes to lysosomes in macrophages, these cells almost exclusively release precursor forms of HYAL1, suggesting that lysosomal exocytosis significantly contributes to HYAL1 secretion only in differentiated osteoclasts. Interestingly, the osteoclastogenesis-dependent mode of secretion of mature HYAL1 develops in parallel with the appearance of secretory lysosomes in osteoclasts. Coupled with the similarities detected between HYAL1 and the secretory lysosome component cathepsin K (i.e. their striking upregulation upon osteoclast differentiation and their perfect co-distributions in all of our fractionation experiments), it is likely that HYAL1 resides, at least partly, in those specialized lysosomes. HYAL1 does not use classical trafficking mechanisms to reach this site though. The sorting of HYAL1 is mainly Man-6-P-independent, in contrast to cathepsin K and TRAP [7,17]. In addition, the mannose receptor-mediated recapture mechanism that drives HYAL1 internalization in precursor macrophages [26] is downregulated upon differentiation into osteoclasts. These observations indicate that osteoclasts promote the extracellular accumulation of secreted HYAL1, possibly resulting in its concentration in the resorption lacuna (often referred to as an “extracellular lysosome”) upon polarization of these cells on bone. Importantly, our findings also consolidate the view that the subcellular trafficking of lysosomal hydrolases in osteoclasts relies, in part, on mannose 6-phosphate-independent sorting pathways. Indeed, it has also been demonstrated that the subcellular trafficking of the aspartic protease cathepsin D, which is mostly found in a population of osteoclast lysosomes with low cathepsin K content and, to a lesser extent, in cathepsin K-enriched secretory lysosomes, is not affected by the disruption of the Man-6-P sorting pathway [7]. Although we cannot exclude that a common alternative mechanism drives the lysosomal sorting of both cathepsin D and HYAL1 in osteoclasts, the lack of sequence similarities between these hydrolases, and the fact that several alternative transport receptors have been found for cathepsin D in other cell types [41–44], suggests that the biogenesis of lysosomes/secretory lysosomes in osteoclasts, and therefore the bone resorption activity of those cells, might be controlled by several transport mechanisms.

Variations of expression of the mannose receptor have been reported in several situations, e.g. it is downregulated in macrophages upon incubation with pathogens or lymphokines, and upregulated by dexamethasone, prostaglandins and IL-4 in macrophages [45–49]. However, the relationship between these changes of expression and biological function(s) remains poorly understood. Osteoclasts differentiate from macrophages, which themselves derive from monocytes. Interestingly, while macrophages contain a large amount of mannose receptors, circulating monocytes are mostly devoid of this receptor [50]. Knowing that the mannose receptor plays a predominant role in the capture of extracellular/circulating acid hydrolases (*N*-glycosylated) [26,45,51–53], this observation suggests that, in macrophages, the acquisition of the lysosomal hydrolytic arsenal may significantly rely on this receptor. In accordance with this view, it has been documented that the lysosomal degradation activity of liver sinusoidal cells, which express high levels of the mannose receptor, decreases by almost 50 percent when the mannose receptor is knocked-out [54]. We now report that the mannose receptor is strikingly downregulated upon differentiation of macrophages into osteoclasts. It is well known that osteoclasts specialize in the degradation of extracellular bone matrix through several mechanisms, including the formation of secretory lysosomes, the upregulation of a subset of lysosomal hydrolases and membrane proteins (including the vATPase, cathepsin K and TRAP), the cell polarization when in contact with bone, and the formation of a sealed extracellular lacuna in which secretory lysosomes release their content. Our findings reveal that osteoclastogenesis promotes bone matrix degradation in this lacuna in two additional ways: by upregulating the glycosidase that is endowed with the highest HA depolymerization activity of all hyaluronidases, i.e. HYAL1, and through the downregulation of the cell surface mannose receptor that would otherwise recapture exocytosed hydrolases. Our results also suggest that, by contrast to macrophages, lysosome biogenesis in differentiating osteoclasts does not heavily rely on cell surface mannose receptors.

Taken together with the reports that, depending on its size and receptors, HA can influence osteoclast and osteoblast function, the selective upregulation of HYAL1 in osteoclasts as well as its subcellular localization and secretion by those cells support the view that HYAL1 is a key actor of bone metabolism.

## Materials and Methods

### Osteoclast differentiation

The mouse macrophage cell line RAW264.7 was obtained from ATCC (TIB-71^™^), cultured in DMEM (Lonza) containing 10% of inactivated FBS (Sigma-Aldrich) and supplemented with 100 U/mL of penicillin and 100 μg/mL of streptomycin (Lonza). Mycoplasma contamination was checked using the MycoAlert^™^ PLUS Mycoplasma Detection Kit (Lonza). To generate osteoclasts from these cells, they were treated with 20 ng/mL of RANKL (R&D Systems) and the medium was changed every other day. Cells were collected at day 0 (prior to treatment), as well as after 2 and 5 days of RANKL treatment.

BMM were prepared as described [7] with slight modifications. Briefly, BMM were harvested from femurs of 2–5 month old male C57BL/6 mice euthanized with CO_2_ and cultured for 6 days in Minimum Essential Medium Eagle-alpha modification (α-MEM, Lonza) containing 10% of inactivated FBS (Gibco), 1/10^th^ volume of L929 cell culture supernatant which contains M-CSF [55], 2 mM of glutamine (Lonza), 100 U/mL of penicillin and 100 μg/mL of streptomycin (Lonza). After trypsinization and plating, the differentiation was induced by adding 20 ng/mL of RANKL to the medium, which was replaced every other day. *Hyal1* -/- mice (B6.129X1-*Hyal1*^*tm1Stn*^/Mmucd) purchased from MMRRC (Mutant Mouse Resource Research Centers, USA) were raised in our laboratory and backcrossed for 9 generations on a C57BL/6 genetic background. This study required the use of 9 mice. All experimental procedures were approved by the Animal Ethics Committee of the University of Namur.

### Quantitative PCR (qPCR)

Total RNA was extracted from cells and reverse transcribed into cDNA using the High Pure RNA Isolation Kit (Roche) and the RevertAid H Minus First Strand cDNA Synthesis Kit (Thermo Fisher Scientific) according to the manufacturer's protocol, respectively. qPCR was performed with the FastStart Universal SYBR Green Master (Roche), using a 7300 Real-Time PCR System (Applied Biosystems) and gene expression level was normalized using the housekeeping gene *Gapdh*. Fold change (2^-ΔΔCT^) between macrophages and osteoclasts samples for each gene was calculated from the average ΔCT values obtained in 3 independent experiments (including 2 replicates for each gene). The sequences of the primers used are listed in S2 Table.

### Western blotting

The western blotting experiments were performed as previously described [26]. The following antibodies were used: mouse monoclonal anti-GAPDH (1:4 000 dilution, G8795, Sigma-Aldrich), anti-HYAL1 (1:1 000 dilution, 1D10, produced by hybridoma cells generously provided by B. Triggs-Raine, University of Manitoba, Winnipeg, Canada) and anti-cathepsin K (1:1 000 dilution, MAB3324, Millipore), as well as goat polyclonal anti-TRAP (1:1 000 dilution, SC-30833, Santa Cruz Biotechnology). When conditioned media were prepared, the cells were cultured for 5 h in serum-free medium prior to lysis of the cells in PBS—Triton X-100 1% supplemented with protease inhibitors (cOmplete, mini protease inhibitors cocktail, Roche). When indicated, cells were incubated for 24 h with either 15 mg/mL of mannan (Sigma-Aldrich) or 5 mM of Man-6-P (Sigma-Aldrich). In Fig 5C, cell extracts and conditioned media were treated with PNGase F (New England Biolabs) according to the manufacturer's instructions. Of note, the specificity of the anti-HYAL1 antibody was validated by an absence of signal in osteoclasts differentiated from BMM of *Hyal1* -/- mice (S1A Fig). HYAL1 signals were quantified using the ImageJ (Rasband, W.S., ImageJ, U. S. National Institutes of Health, Bethesda, Maryland, USA, http://imagej.nih.gov/ij/, 1997–2015).

### Enzymatic assays

The HA-degrading activity of HYAL1 was analyzed by "native" and "renatured protein" zymography as detailed in Puissant et al. [26]. Signals were quantified using the ImageJ software.

The enzymatic activity of lysosomal acid hydrolases and ER marker alkaline α-glucosidase was measured with the following 4-methylumbelliferyl-coupled specific substrates (Sigma-Aldrich): 4-methylumbelliferyl-β-D-galactopyranoside (β-galactosidase), 4-methylumbelliferyl-β-D-glucuronide hydrate (β-glucuronidase), 4-methylumbelliferyl-*N*-acetyl-β-D-glucosaminide (β-hexosaminidase) and 4-methylumbelliferyl-α-D-glucopyranoside (alkaline α-glucosidase). The samples were incubated at 37°C with 5 mM of substrate in a 50 mM citrate buffer, pH 4.5 containing 0.05% Triton X-100, except for the alkaline α-glucosidase activity assay. In this case, the reaction was conducted using 1 mM of substrate diluted in a 0.1 M glycine-NaOH solution (pH 9) containing 0.05% Triton X-100. After 6 h of reaction, a 0.1 M glycine-NaOH solution (pH 10.3) was added to stop the enzymatic activities and the fluorescence was measured at 495 nm.

### HYAL1 endocytosis assays

RAW264.7-derived osteoclasts and their precursor cells were incubated for 2 h at 37°C in the presence of 4 μg/mL of rhHYAL1, diluted in DMEM containing FBS and supplemented, when indicated, with 75 μM of leupeptin and pepstatin A (Sigma-Aldrich). A 15 min chase period in the absence of rhHYAL1 was then conducted and the cells were lysed in PBS—1% Triton X-100 containing protease inhibitors. The amount of HYAL1 endocytosed by the cells was assessed by renatured protein zymography as described above.

To quantify endocytosis levels more precisely, RAW264.7 macrophages and derived osteoclasts plated in 12-well plates were incubated at 37°C for 5, 10 or 30 min in DMEM containing FBS and rhHYAL1 labeled with ^125^Iodine, as described in Puissant et al., 2014 [26]. To test the impact of mannan on HYAL1 endocytosis, the cells were pre-treated for 5 min with PBS containing 1% of BSA and 10 mg/mL of mannan, then incubated with ^125^I-HYAL1 for the indicated periods of time in the presence of 20 mg/mL of mannan. The cells were then washed with PBS and cell proteins bound to the cell surface stripped with a 0.5 M NaCl/0.2 M acetic acid solution, pH 3.5, for 30 sec at 4°C. Lastly, the cells were lysed in 0.1 M NaOH and the intracellular radioactivity counted using a Beckman counter (Beckman Coulter LS 6500+).

### Subcellular fractionation

A slightly modified version of the differential fractionation protocol described by de Duve et al. [35] was used to separate RAW264.7-derived osteoclasts into five different subcellular fractions: nuclear (N), heavy mitochondrial (M), light mitochondrial (L), microsomal (P), and soluble (S). The protocol is detailed in Puissant et al., 2014 [26], except for the following modification: the M and L fractions were prepared separately, as initially described [35].

An adapted version of the protocol described in Green et al. [56] was then used to further separate some organelles in a self-forming Percoll^™^ density gradient. Briefly, the M, L and P fractions were pooled and loaded on top of an 18% Percoll^™^ solution (18% v/v Percoll^™^ [Pharmacia], 0.25 M sucrose, 2 mM EDTA and 10 mM Tris-HCl, pH 7.4). After centrifugation at 59 000 × *g* in a SW55Ti rotor (Beckman Coulter) for 40 min at 4°C, the gradient was divided into seven fractions from top to bottom. As a control of the formation of the density gradient, the refractive index of each fraction was measured with a refractometer. The activity of marker enzymes was measured in all collected fractions as described above. For western blotting and zymography assays, an aliquot of each Percoll^™^ gradient fraction was incubated with 1% Triton X-100 for 30 min at 4°C, then centrifuged for 30 min at 200 000 x *g* in a TLA-100.3 rotor (Beckman Coulter) at 4°C to remove the Percoll^™^ beads.

When indicated the cells were pre-treated for 24 h with 2 μg/mL of U18666A (Millipore), a molecule that inhibits the lysosomal export of cholesterol [36,57], prior to fractionation of an M+L+P pooled fraction in a preformed linear sucrose density gradient (1.04–1.26 g/mL). Isopycnic centrifugation was performed at 144 000 x *g* for 16 h in a SW55Ti rotor. Nine fractions were collected and assayed for marker enzyme activities and presence of HYAL1 and cathepsin K as described above.

### CI-MPR affinity chromatography

As previously described [26], the presence of Man-6-P moieties on HYAL1 and cathepsin K was assessed using CI-MPR affinity chromatography [40]. Briefly, cell lysates and conditioned media (collected after a 5 h culture period in the absence of serum) were loaded on 1 mL columns made of CI-MPRs immobilized on a sepharose 4B matrix (a generous gift from P. Lobel, Center for Advanced Biotechnology and Medicine, Piscataway, USA). After washes and elution of non-specifically bound proteins with 5mM of glucose 6-phosphate (Gluc-6-P, Sigma-Aldrich), the mannose 6-phosphorylated proteins were eluted with 5 mM of Man-6-P (Sigma-Aldrich). The collected fractions were pooled two by two, precipitated with trichloroacetic acid and resolved by SDS-PAGE to detect HYAL1 and cathepsin K using western blotting.

## Supporting Information

S1 Fig HYAL1 is overexpressed and secreted by BMM-derived osteoclasts. (A) To check the specificity of the anti-HYAL1 antibody used throughout this work, BMM were isolated from wild-type (WT) or HYAL1 deficient (*Hyal1* -/-) mice. These cells were then cultured in the presence of M-CSF for 6 days, and subsequently differentiated into osteoclasts by the addition of RANKL. WT and *Hyal1* -/- osteoclast lysates were then analyzed by western blotting, which demonstrates the absence of signal in the knockout cells. (B) The expression of HYAL1, cathepsin K, TRAP and GAPDH was detected by western blotting at day 2 and day 5 of the differentiation process of BMM into osteoclasts. Precursor and mature forms of the enzyme are pointed by closed and open arrowheads, respectively. (C) The activity of HYAL1 was analyzed in BMM and osteoclasts derived from these cells by renatured and native protein zymography. Closed and open arrowheads point to precursor and mature forms, respectively. (D) Measurement of the enzymatic activity of β-hexosaminidase and β-glucuronidase upon osteoclastogenesis of BMM. Results are shown as fold change in osteoclasts relative to their precursor cells (mean ± SD of *n* = 3 independent experiments). (E) After 5 h of incubation in serum-free conditions, the secretion of HYAL1, cathepsin K and TRAP was analyzed by western blotting. The cell integrity was confirmed by the absence of the cytosolic protein GAPDH in the conditioned medium (concentrated 4-fold). The 65 kDa secreted form, 52 kDa precursor form and 48 kDa cleaved form of HYAL1 are highlighted by asterisk, closed and open arrowheads, respectively. The precursor and mature forms of cathepsin K and TRAP are pointed by closed and open arrowheads, respectively.
(TIF)

S1 Table Fold change in mRNA expression levels of hyaluronidases and lysosomal hydrolases in osteoclasts collected at day 2 or 5 of their differentiation process, compared to BMM precursor macrophages (day 0). (DOCX)

S2 Table Sequences (5'-3') of the primers used in qPCR. (DOCX)
