# Supplementary figures and images for: Monocytes/Macrophages Upregulate the Hyaluronidase HYAL1 and Adapt Its Subcellular Trafficking to Promote Extracellular Residency upon Differentiation into Osteoclasts
Source: PLoS One. 2016 Oct 18;11(10):e0165004. doi: 10.1371/journal.pone.0165004 (PMC5068775; doi:10.1371/journal.pone.0165004)

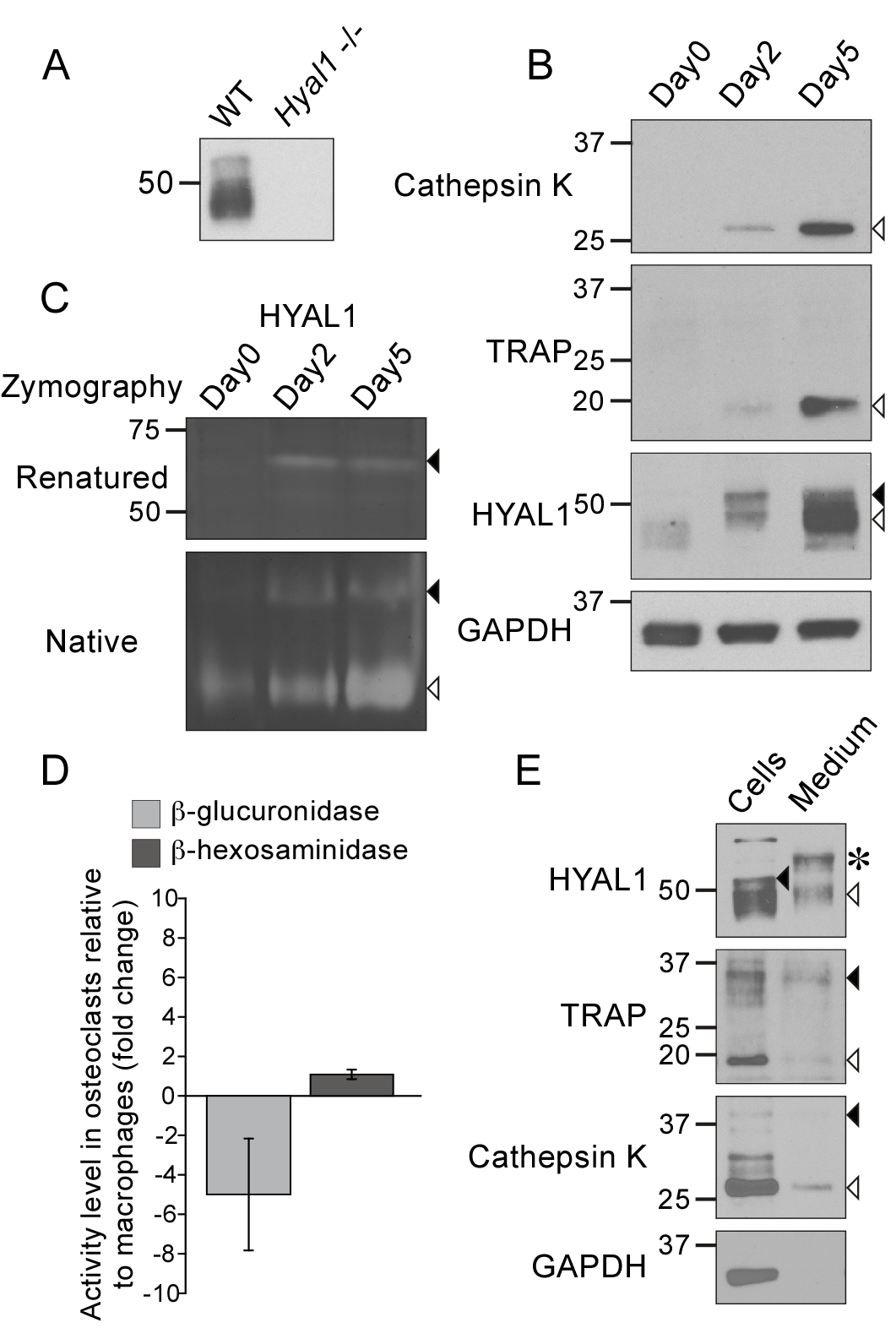

Supplement: S1 Fig — (A) To check the specificity of the anti-HYAL1 antibody used throughout this work, BMM were isolated from wild-type (WT) or HYAL1 deficient (Hyal1 -/-) mice. These cells were then cultured in the presence of M-CSF for 6 days, and subsequently differentiated into osteoclasts by the addition of RANKL. WT and Hyal1 -/- osteoclast lysates were then analyzed by western blotting, which demonstrates the absence of signal in the knockout cells. (B) The expression of HYAL1, cathepsin K, TRAP and GAPDH was detected by western blotting at day 2 and day 5 of the differentiation process of BMM into osteoclasts. Precursor and mature forms of the enzyme are pointed by closed and open arrowheads, respectively. (C) The activity of HYAL1 was analyzed in BMM and osteoclasts derived from these cells by renatured and native protein zymography. Closed and open arrowheads point to precursor and mature forms, respectively. (D) Measurement of the enzymatic activity of β-hexosaminidase and β-glucuronidase upon osteoclastogenesis of BMM. Results are shown as fold change in osteoclasts relative to their precursor cells (mean ± SD of n = 3 independent experiments). (E) After 5 h of incubation in serum-free conditions, the secretion of HYAL1, cathepsin K and TRAP was analyzed by western blotting. The cell integrity was confirmed by the absence of the cytosolic protein GAPDH in the conditioned medium (concentrated 4-fold). The 65 kDa secreted form, 52 kDa precursor form and 48 kDa cleaved form of HYAL1 are highlighted by asterisk, closed and open arrowheads, respectively. The precursor and mature forms of cathepsin K and TRAP are pointed by closed and open arrowheads, respectively. (TIF) [file pone.0165004.s001.tif]
