# Supplementary material for: Monocytes/Macrophages Upregulate the Hyaluronidase HYAL1 and Adapt Its Subcellular Trafficking to Promote Extracellular Residency upon Differentiation into Osteoclasts
Source: PLoS One. 2016 Oct 18;11(10):e0165004. doi: 10.1371/journal.pone.0165004 (PMC5068775; doi:10.1371/journal.pone.0165004)
Supplement: S1 Table — (DOCX) [file pone.0165004.s002.docx]

**S1 Table. Fold change in mRNA expression levels of hyaluronidases and lysosomal hydrolases in osteoclasts collected at day 2 or 5 of their differentiation process, compared to BMM precursor macrophages (day 0).**

| Protein / Gene name | Fold change | |
| --- | --- | --- |
|  | **Day 2 / Day 0** | **Day 5 / Day 0** |
| Cathepsin K / *Ctsk* | 270.22 | 582.05 |
| TRAP / *Acp5* | 1192.69 | 3046.63 |
| HYAL1 / *Hyal1* | 7.84 | 10.90 |
| HYAL2 / *Hyal2* |  | 3.07 |
| HYAL3 / *Hyal3* |  | 5.52 |
| β-glucuronidase / *Gusb* |  | 1.22 |
| β-hexosaminidase α / *Hexa* |  | 2.01 |
| β-hexosaminidase β / *Hexb* |  | 2.86 |
| Cathepsin D / *Ctsd* |  | -1.36 |
| β-galactosidase / *Glb1* |  | 3.68 |
| β-glucocerebrosidase / *Gba* |  | 1.48 |
| β-mannosidase / *Manba* |  | 1.86 |
