# Supplementary material for: Monocytes/Macrophages Upregulate the Hyaluronidase HYAL1 and Adapt Its Subcellular Trafficking to Promote Extracellular Residency upon Differentiation into Osteoclasts
Source: PLoS One. 2016 Oct 18;11(10):e0165004. doi: 10.1371/journal.pone.0165004 (PMC5068775; doi:10.1371/journal.pone.0165004)
Supplement: S2 Table — (DOCX) [file pone.0165004.s003.docx]

**S2 Table. Sequences (5'-3') of the primers used in qPCR.**

| Protein / Gene name | Forward | Reverse |
| --- | --- | --- |
| β-galactosidase / *Glb1* | CCGGATACCCCGCTTCTACT | TCACGGTCCCCAGAAAACTC |
| β-glucocerebrosidase / *Gba* | ATTTGGAGGCGCCATGACA | AGTCGTTAGGGGTGTCAGCA |
| β-glucuronidase / *Gusb* | ATAAGACGCATCAGAAGCCG | ACTCCTCACTGAACATGCGA |
| β-hexosaminidase α / *Hexa* | TTCAAGCAGCTGGAGTCCTTCT | CCCTTGTCATAATCAGAGACGATGT |
| β-hexosaminidase β / *Hexb* | ACCACTTTTCCTGAGCTAAGCAA | GGACATCGTTTGGTGTATAGACATG |
| β-mannosidase / *Manba* | AGCCCAACCAACTACCACTT | CAACCAAACAAAGGGAGCGAC |
| Cathepsin D / *Ctsd* | TTCGTCCTCCTTCGCGATT | CTCCGTCATAGTCCGACGGATA |
| Cathepsin K / *Ctsk* | gaagaagactcaccagaagcag | Tccaggttatgggcagagatt |
| CI-MPR / *Igf2r* | CTGCAAGAGAGGAGTCAGCA | CGTCAGGACAGACAATCGGA |
| GAPDH / *Gapdh* | CGTGCCGCCTGGAGAA | GATGCCTGCTTCACCACCTT |
| HYAL1 / *Hyal1* | CAGCATGCTCAGAAAGTTTGG | TGAGCAAGGTGGGTAACCAG |
| HYAL2 / *Hyal2* | CGAGGACTCACGGGACTGA | GGCACTCTCACCGATGGTAGA |
| HYAL3 / *Hyal3* | CCGGAGCTCTGGGAGATTC | GCGGCACTCACTCCAATAGTC |
| Mannose receptor / *Mrc1* | agtgatggttctcccgtttcctat | Tgactgcccaccattcttgtttat |
| TRAP / *Acp5* | GGTATGTGCTGGCTGGAAAC | ACGTGGAATTTTGAAGCGCAA |
